# IgA nephropathy featuring massive wire loop-like deposits in two patients with alcoholic cirrhosis

**DOI:** 10.1186/s12882-017-0769-1

**Published:** 2017-12-13

**Authors:** Daisuke Takada, Keiichi Sumida, Akinari Sekine, Ryo Hazue, Masayuki Yamanouchi, Tatsuya Suwabe, Noriko Hayami, Junichi Hoshino, Naoki Sawa, Kenmei Takaichi, Takeshi Fujii, Kenichi Ohashi, Yoshifumi Ubara

**Affiliations:** 1 Nephrology Center, Toranomon Hospital Kajigaya, 1-3-1, Takatsu, Kawasaki, Kanagawa 213-8587 Japan; 2 Department of Pathology, Toranomon Hospital, Tokyo, Japan; 3 The Okinaka Memorial Institute for Medical Research, Toranomon Hospital, Tokyo, Japan; 4 Department of Pathology, Yokohama City University, Graduate School of Medicine, Yokohama, Japan

**Keywords:** Wire loop-like subendothelial deposit, Alcoholic cirrhosis, IgA nephropathy

## Abstract

**Background:**

Various renal manifestations are known to develop in patients with liver disease, including chronic hepatitis and cirrhosis.

**Case presentation:**

We evaluated renal disease in two 47-year-old Japanese men with liver cirrhosis and chronic alcoholism for 34 years and 27 years, respectively. Renal biopsy demonstrated massive wire loop-like deposits in the subendothelial space of the glomerular basement membrane and in the mesangium. However, immunofluorescence was only positive for IgA and C3, and electron microscopy did not reveal any organized structures in the electron-dense deposits. IgA nephropathy was diagnosed, although the features were different from primary IgA nephropathy. Both patients had portosystemic shunts associated with liver cirrhosis. Their renal deposits and proteinuria resolved completely after 1 year of steroid therapy.

**Conclusion:**

Alcohol abuse may have contributed to development of secondary IgA nephropathy in these two patients, probably via their portosystemic shunts.

## Background

Various renal manifestations are known to develop in patients with liver disease, including chronic hepatitis and cirrhosis. Three types of nephropathy have been reported in patients with hepatitis C virus (HCV) infection, including IgG-dominant nephropathies (such as membranous glomerulonephritis: MGN), IgA-dominant nephropathies (such as IgA nephropathy), and IgM-dominant nephropathies (such as type 1 membranoproliferative glomerulonephritis (MPGN), which corresponds to cryoglobulinemic glomerulopathy). The most common form of HCV-related renal disease is cryoglobulinemic glomerulopathy, which is closely associated with hypocomplementemia, liver cirrhosis, and elevation of serum HCV RNA [1–3]. On the other hand, the best-known renal complication of hepatitis B virus (HBV) infection is MGN. Type 1 MPGN with IgG dominant staining has also been reported in patients with HBV surface antigen positivity [3]. Although an animal study has shown that IgA nephropathy can be induced by alcohol abuse [4], glomerulonephritis specific to alcoholic liver disease has not been clearly defined and is categorized with cirrhosis-related secondary IgA nephropathy. In patients with cirrhosis-related IgA nephropathy, renal histologic changes are generally reported to be mild, consisting of slight to moderate mesangial expansion with or without mesangial hypercellularity [5].

Here we report two patients with alcoholic cirrhosis who developed IgA nephropathy featuring massive subendothelial deposits, and we document the response of their renal histology and proteinuria to steroid therapy.

## Case presentations

### Case 1

A 47-year-old Japanese man was admitted to our hospital for evaluation of renal disease in March 2009. Liver dysfunction was initially detected in December 2007. He consulted a local clinic in February 2008 because of generalized edema and weight gain from 70 kg to 78 kg. Proteinuria (4+) was also detected. His anasarca was treated with furosemide (150 mg daily) and restriction of salt and fluid intake (to 6 g of salt and 1000 mL of water daily). He had a history of drinking 1500 mL of beer daily for 27 years from the age of 20. On admission, he was 173 cm tall and weighed 70.6 kg. His blood pressure was 138/70 mmHg and he had bilateral lower limb edema. Laboratory findings are shown in Table 1. Total protein was 5.5 g/dL, albumin was 2.2 g/dL, urea nitrogen was 17 mg/dL, and creatinine (Cre) was 0.9 mg/dL. In addition, 24-h urinary protein excretion was 3.9 g and the urine sediment contained numerous erythrocytes per high-power field.

**Table 1:** Laboratory findings of case 1 before and after 1 year of steroid therapy

	Before	After 1 year	Normal range
White blood count, /μl	4600	6600	3400–9200
Red blood cell, ×10*4/μl	4.2	2.61	400–566
Hemoglobin, g/dl	13.4	9	13.0–17.0
Hematocrit, %	41.3.6	28	38.2–50.8
Platelet, ×10*4/μl	17.6	13	14.1–32.7
Total protein, g/dl	5.5	6.8	6.9–8.4
Albumin, g/dl	2.2	2.7	3.9–5.2
Total bilirubin, mg/dl	1.5	2.6	0.3–1.1
AST, IU/l	60	70	13–33
ALT, IU/l	38	36	8–42
LDH, IU/l	204	250	119–229
CPK, IU/l	62	42	62–287
ALP, IU/l	232	304	117–350
γGTP, IU/l	140	358	9–109
LAP	54	63	21–42
ChE	184	143	220–495
T-Chol	174	210	120–240
TG	76	151	30–150
NH3	91	62	6–51
HbA1c	4.7		4.6–6.2
UN, mg/dl	17	17	8–12
Creatinine, mg/dl	0.9	1.1	0.65–1.06
eGFR	72.1	57.9	>100
Urinary acid, mg/dl	6.5	7.7	2.5–7.0
Na, mmol/l	140	139	139–146
K, mmol/l	4.2	4	3.7–4.8
Cl, mmol/l	104	102	101–108
APTT, sec	41.7	31.1	27–40
PT,%	79.4	53.2	>75
INR,	1.15	1.3	
ICG15R(%)	58	32	<10
IgG, mg/dl	740	947	870–1700
IgA, mg/dl	1110	845	110–410
IgM, mg/dl	162	190	35–220
IgE, mg/dl			<173
C3, mg/dl	66	78	86–160
C4, mg/dl	13	15	17–45
CH50, U/ml	31	35	30–50
ANA	negative	negative	
anti ds-DNA	negative	negative	
anti RNP	negative	negative	
anti mitochondria	negative	negative	
Cryoglobulin	negative	negative	
HCV	negative	negative	
HBV	negative	negative	
CRP, mg/dl	0.1	0.1	0.0–0.3
Urinalysis			
Sediment			
RBC /HPF	many	1 to 4	<1
WBC /HPF	6–10	–	<1
Cast	None	None	<1
protein, g/day	3.94	0.04	<0.1
Glucose	negative	negative	negative
NAG, IU/day	14.3	8.9	0.8–5.0
α1 MG, mg/day	8.1	2.1	0.6–8.8
β2 MG, mg/day	46	30	14–329

Computed tomography and ultrasonography showed enlargement of the paraumbilical veins (suggesting a portosystemic shunt), as well as hepatosplenomegaly and ascites. Small esophageal varices with a negative red color sign were detected by endoscopy.

Alcoholic cirrhosis was diagnosed from the 27-year history of alcohol abuse and negative test results for HBV and HCV infection. Renal biopsy was performed to evaluate the cause of his renal disease.

### Case 2

A 47-year-old Japanese man was admitted to our hospital for evaluation of renal disease in May 2013. Edema of the lower limbs was initially noted in August 2012. He consulted a local clinic in February 2013 with generalized edema and weight gain to 113 kg. Proteinuria was detected (5.2 g daily). His anasarca was treated with furosemide (100 mg daily) and restriction of salt and fluid intake (to 6 g of salt and 700 mL of water daily), achieving weight reduction to 90.0 kg. He had a history of drinking one-third of a bottle of whisky daily for 34 years starting from 13 years old. On admission, he was 174 cm tall and weighed 90.0 kg, with a blood pressure of 144/88 mmHg and bilateral lower limb edema. Laboratory findings are shown in Table 2. Total protein was 6.0 g/dL, albumin was 2.7 g/dL, urea nitrogen was 23 mg/dL, and Cre was 1.66 mg/dL. In addition, 24-h urinary protein excretion was 1.17 g and the urine sediment contained more than 100 erythrocytes per high-power field.

**Table 2:** Laboratory findings of case 2 before and after 1 year of steroid therapy

	Before	After 1 year	Normal range
White blood count, /μl	10,300	8700	3400–9200
Red blood cell, ×10*4/μl	4.14	3.95	400–566
Hemoglobin, g/dl	12.4	11.1	13.0–17.0
Hematocrit, %	36.6	34.5	38.2–50.8
Platelet, ×10*4/μl	14.7	18.6	14.1–32.7
Total protein, g/dl	6	6.5	6.9–8.4
Albumin, g/dl	2.7	2.8	3.9–5.2
Total bilirubin, mg/dl	0.6	0.5	0.3–1.1
AST, IU/l	65	32	13–33
ALT, IU/l	38	10	8–42
LDH, IU/l	231	234	119–229
CPK, IU/l	118	94	62–287
ALP, IU/l	298	298	117–350
γGTP, IU/l	172	63	9–109
LAP	54		21–42
ChE	204		220–495
T-Chol	172	137	120–240
TG	111	167	30–150
NH3	31	38	6–51
HbA1c	4.9	4.6	4.6–6.2
UN, mg/dl	23	21	8–12
Creatinine, mg/dl	1.66	1.55	0.65–1.06
eGFR	36.8	47.5	
Urinary acid, mg/dl	9.3	6.7	2.5–7.0
Na, mmol/l	144	141	139–146
K, mmol/l	4.2	5	3.7–4.8
Cl, mmol/l	107	110	101–108
APTT, sec	25.9	30.9	27–40
PT,%	99.3	89.5	>75
INR,	1	1	
ICG15R(%)	27		
IgG, mg/dl	958	1195	870–1700
IgA, mg/dl	519	324	110–410
IgM, mg/dl	149	1216	35–220
IgE, mg/dl	88.9		<173
C3, mg/dl	62	89	86–160
C4, mg/dl	17	37	17–45
CH50, U/ml	34	42	30–50
ANA	negative	negative	
anti ds-DNA	negative	negative	
anti RNP	negative	negative	
anti mito	negative	negative	
Cryoglobulin	negative	negative	
HCV	negative	negative	
HBV	negative	negative	
CRP, mg/dl	0	0	0.0–0.3
Urinalysis			
Sediment			
RBC, /HPF	many	1 to 4	<1
WBC, /HPF	1–4	–	<1
Cast	None	None	<1
protein, g/gCre	1.17	0.01	
Glucose	negative	negative	negative
NAG, IU/day	14.3	8.9	0.8–5.0
α1 MG, mg/day	8.1	0.4	0.6–8.8
β2 MG, mg/day	14	19	14–329

Computed tomography showed gastric varices with anastomoses to the left renal vein, as well as cirrhosis and splenomegaly.

This patient was also diagnosed as having alcoholic cirrhosis due to his 34-year history of alcohol abuse and negative tests for HBV and HCV infection. As with case 1, renal biopsy was performed to evaluate his renal disease.

### Findings at the first renal biopsy

#### Case 1

**Fig. 1:**
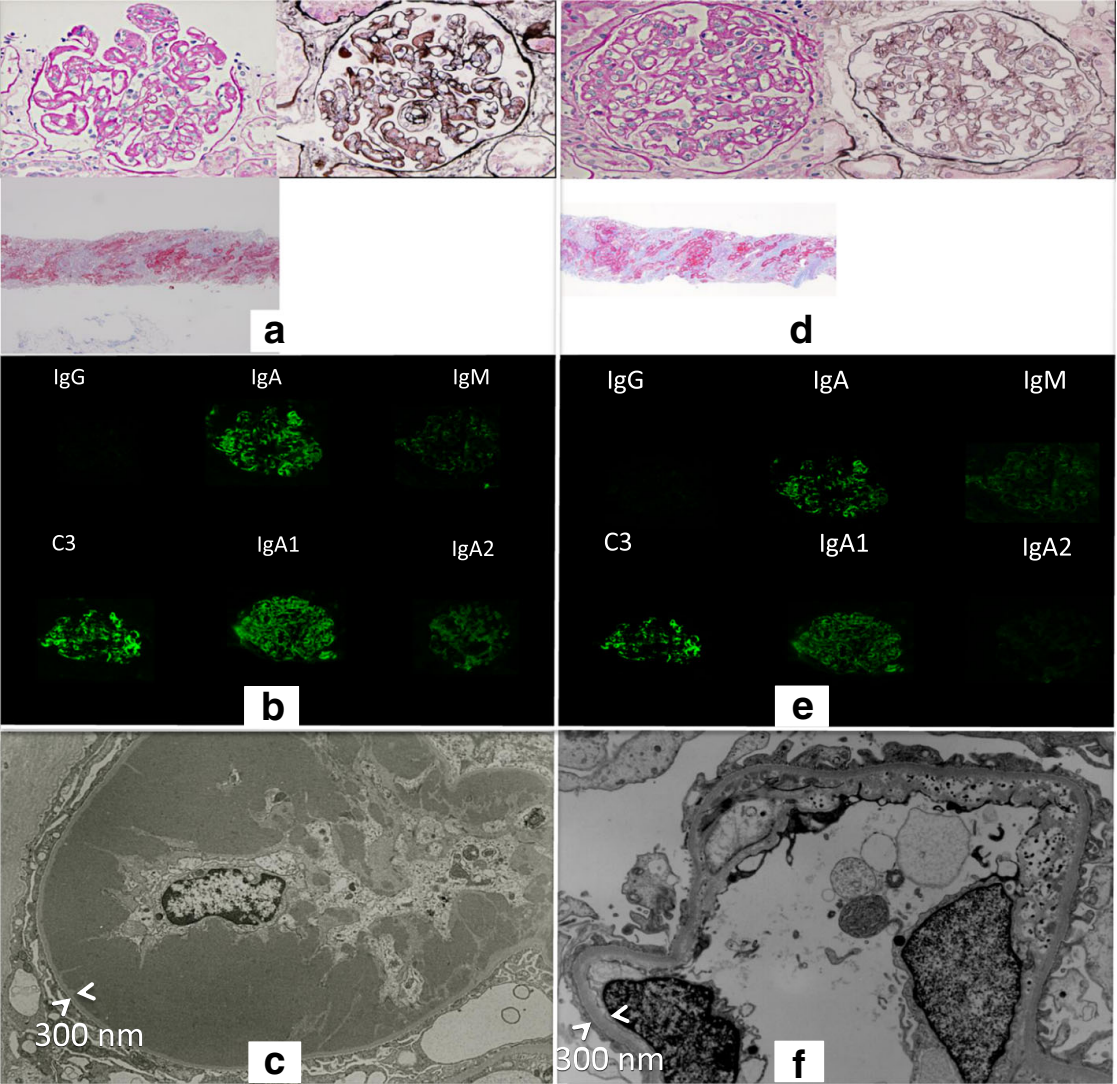
**a**: Case 1: Light microscopy (LM) of the first renal biopsy specimen shows massive subendothelial wire loop-like deposits of amorphous material in the glomerular basement membrane (GBM) and similar deposits the mesangium. **b**: Immunofluorescence (IF) is positive for IgA(IgA1 > IgA2) and C3, predominantly in the mesangium and GBM. **c**: Electron microscopy (EM) shows huge subendothelial electron-dense deposits (EDD) without an organized internal structure in the GBM and similar deposits in the mesangium. **d**: LM of the second renal biopsy specimen demonstrates marked improvement of the glomerular changes. **e**: IF shows weaker staining for IgA and C3 compared to that seen in the first biopsy. **f**: EM reveals disappearance of the massive EDD

Light microscopy (LM) of the biopsy specimen revealed global sclerosis in 1 out of 25 glomeruli. In the other glomeruli, there was slight to moderate expansion of the mesangial matrix and mesangial cell proliferation, as well as massive wire loop-like amorphous subendothelial deposits in the glomerular basement membrane (GBM) and endothelial cell swelling (Fig. 1a). Immunofluorescence (IF) was positive for IgA (IgA1 > IgA2) and C3, predominantly in the mesangium and GBM, while there was no immunostaining for IgG, IgM, C4, and C1q (Fig. 1b). Electron microscopy (EM) showed massive electron-dense subendothelial deposits without any organized internal structure in the GBM and similar deposits in the mesangium (Fig. 1c). IgA nephropathy was diagnosed, although the renal histology differed from that of primary IgA nephropathy.

#### Case 2

**Fig. 2:**
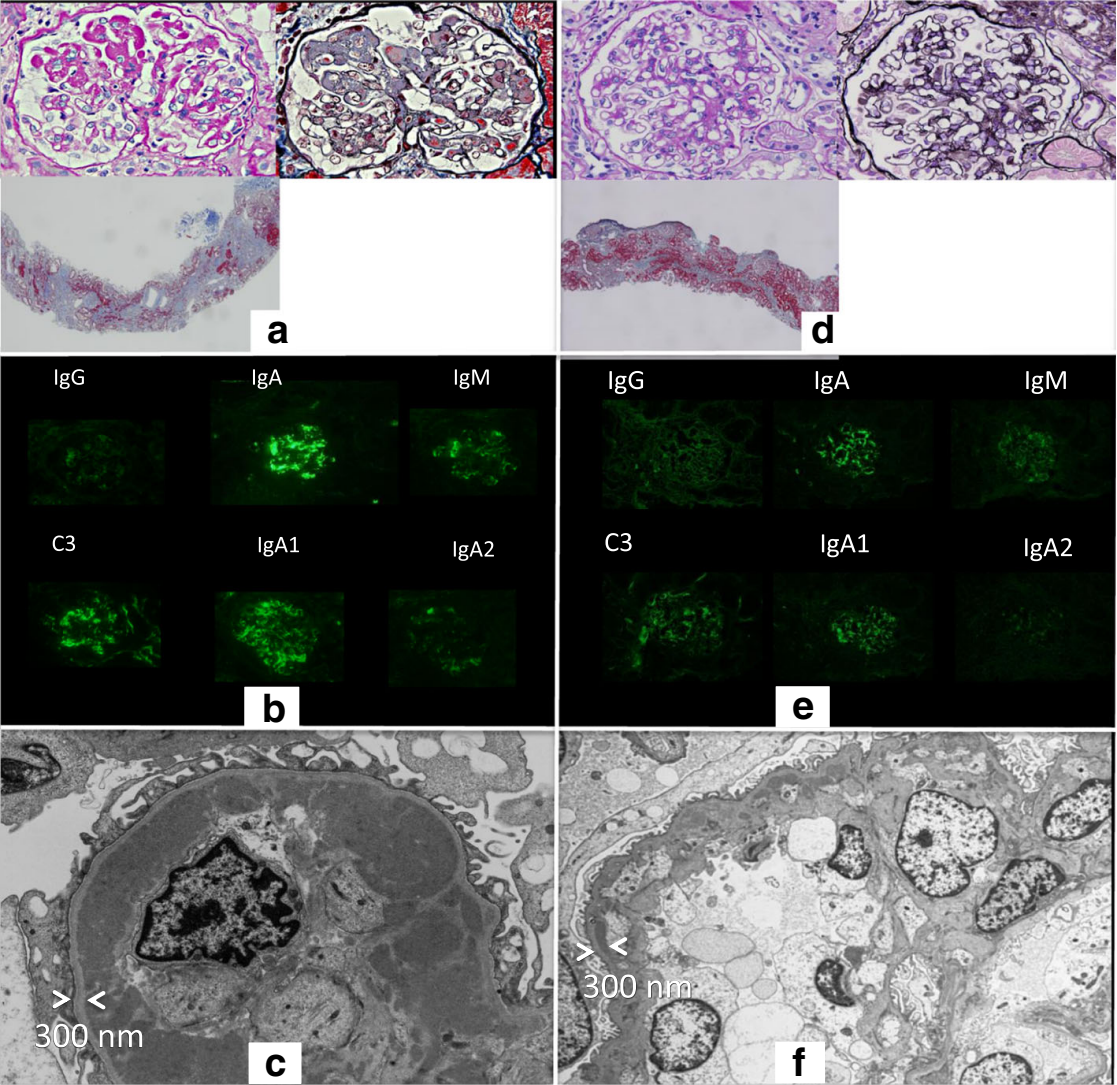
**a**: Case 2: LM of the first renal biopsy specimen demonstrates massive wire loop-like deposits of amorphous material similar to those seen in case 1. **b**: IF displays similar findings to case 1. **c**: EM reveals massive deposits similar to those seen in case 1. **d**: LM of the second renal biopsy specimen shows marked improvement of the glomerular changes. **e**: IF displays weaker staining for IgA and C3 compared to the first biopsy. **f**: Massive EDD are no longer shown by EM

LM revealed global sclerosis in 8 out of 24 glomeruli. The remaining glomeruli showed similar features to those noted in case 1 (Fig. 2a), and IF findings were also similar to those seen in case 1 (Fig. 2b). Furthermore, EM showed similar massive deposits to those detected in case 1 (Fig. 2c). Accordingly, atypical IgA nephropathy was diagnosed in this patient, as in case 1.

### Clinical course

#### Case 1

Treatment was started with intravenous methylprednisolone (0.5 g daily for three days), followed by oral prednisolone (PSL) at a dose of 30 mg on alternate days. PSL was tapered by 5 mg every two months, and was discontinued after 1 year. An angiotensin II receptor blocker (olmesartan at 40 mg/day) was administered to treat hypertension. Six months after starting PSL, proteinuria was reduced to less than 0.1 g daily. Although complete remission of proteinuria was maintained after discontinuation of PSL, renal dysfunction persisted. Accordingly, a second renal biopsy was performed for re-evaluation of this patient’s renal disease at 1 year after the first biopsy.

#### Case 2

Treatment was initiated with the same regimen as that used in case 1 and was discontinued after 1 year. Olmesartan (40 mg/day) was also administered for hypertension. Three months after starting PSL, proteinuria was reduced to less than 0.1 g daily and complete remission of proteinuria was maintained after discontinuation of PSL. However, renal dysfunction also persisted in this patient, so renal biopsy was repeated for re-evaluation of his renal disease after 1 year.

### Findings at the second renal biopsy

#### Case 1

LM of the biopsy specimen revealed global sclerosis in 3 out of 14 glomeruli, while the remaining glomeruli showed marked improvement compared with the initial findings (Fig. 1d). IF demonstrated weaker IgA and C3 staining compared with that at the time of the first biopsy (Fig. 1e), while EM revealed disappearance of the massive subendothelial and mesangial electron-dense deposits (Fig. 1f).

#### Case 2

LM of the biopsy specimen displayed global sclerosis in 5 out of 23 glomeruli, along with marked improvement of the changes in the remaining glomeruli (Fig. 2d). As in case 1, IF showed weaker staining for IgA and C3 compared to the first biopsy (Fig. 2e), while EM revealed elimination of the massive subendothelial and mesangial electron-dense deposits (Fig. 2f).

## Discussion

Alcohol abuse is a very common clinical problem, with an estimated lifetime prevalence of 18% among adults in the United States, and a report in 2010 estimated that alcohol-related cirrhosis was responsible for 493,300 deaths worldwide (1% of all deaths) [6]. In 1942, Horn et al. first identified glomerulonephritis in an autopsy case of liver cirrhosis without diabetes [7]. In 1983, Endo et al. evaluated renal disease in 50 cirrhosis patients at autopsy, detecting membranoproliferative glomerulonephritis (MPGN type l) and mesangial proliferative glomerulonephritis with or without subendothelial deposits. Predominant deposition of IgA was a characteristic finding in these patients. The causes of cirrhosis included alcohol abuse, HBV infection, and non A-non B virus infection, but the relation between an alcoholic etiology and glomerular changes was not reported [8].

In 2009, Kaartinen et al. examined the relation between alcohol intake and IgA nephropathy in 158 patients (95 men), and concluded that moderate alcohol consumption might have a favorable impact on the progression of IgA nephropathy [9]. Koning et al. studied the association between alcohol consumption and chronic kidney disease (CKD) in 5476 persons aged 28 to 75 years, and found and inverse correlation between alcohol consumption and the risk of developing CKD [10].

After reviewing previous reports, Newel concluded that more than 50% of autopsy patients with cirrhosis have mesangial lgA deposits together with complement deposition and elevation of serum IgA. They suggested that spread of circulating immune complexes via portosystemic shunts may be involved in the pathogenesis of renal lesions [11].

Tissandie et al. analyzed IgA-associated serum parameters in 32 patients with compensated or advanced alcoholic cirrhosis. They reported various similarities between primary IgA nephropathy and secondary IgA nephropathy in patients with alcoholic cirrhosis, since features of primary nephropathy such as abnormal glycosylation of IgA1 and soluble CD89-IgA or IgA-IgG complexes were also present in cirrhosis patients. However, IgA1 displayed modified N-glycosylation in patients with alcoholic cirrhosis that was not found in primary IgA nephropathy [12].

IgA nephropathy is the renal disease most frequently associated with prominent mesangial deposition of IgA, and it mainly occurs in Asians and Caucasians. Primary IgA nephropathy is characterized by elevation of serum IgA, the absence of systemic features, and the following renal biopsy findings: LM shows mesangial hypercellularity and an increase of matrix, IF demonstrates IgA staining (often accompanied by C3) in the mesangium and lesser staining along the glomerular capillary walls, and EM typically reveals electron-dense deposits that are primarily limited to the mesangium. Henoch-Schönlein purpura (HSP), also called IgA vasculitis, is a systemic vasculitis associated with palpable purpura, arthritis, abdominal pain, and renal disease. While the renal lesions are similar to those of primary IgA nephropathy, the following points can be used for differentiation. First, LM can show a wide spectrum of glomerular changes in HSP, ranging from isolated mesangial proliferation to severe crescentic glomerulonephritis. In addition, IF may reveal glomerular deposition of IgG, IgM, fibrinogen, and C3 in HSP. EM typically shows electron-dense deposits in the mesangial region, which occasionally extend out into the peripheral capillary loops [13, 14].

There have been two reports showing a close relationship between the existence of a portosystemic shunt and mesangial IgA deposition. Dash et al. performed a prospective study in 200 patients with non-cirrhotic portal fibrosis (NCPF) and bleeding from esophageal varices due to portal hypertension who underwent spleno-renal shunt surgery, after which the portal circulation directly entered the inferior vena cava via the renal vein. In 32% of these patients, nephrotic syndrome developed within five years after surgery. Renal biopsy revealed mesangiocapillary glomerulonephritis in 18.5% of them, while IF showed granular deposits of IgA and C3, and EM demonstrated electron-dense deposits in the mesangium [15]. In addition, Soma also reported three patients who developed type I membranoproliferative glomerulonephritis (MPGN) with IgA deposits from 7 to 13 years after portosystemic shunt surgery [16]. These reports indicate that IgA from the gastrointestinal tract may contribute to the development of IgA nephropathy if it reaches the systemic circulation via a portosystemic shunt.

## Conclusion

We evaluated renal disease in two Japanese men with alcoholic cirrhosis. Renal biopsy revealed IgA nephropathy characterized by glomerular immunostaining for IgA. However, LM showed massive wire loop-like amorphous deposits in the mesangial and subendothelial regions along with endothelial cell swelling, and EM disclosed huge electron-dense deposits without an organized internal structure. Both patients had alcoholic liver cirrhosis with a portosystemic shunt, and their massive renal deposits were completely eliminated after 1 year of steroid therapy. Because renal histologic changes are generally reported to be mild in patients with cirrhosis-related IgA nephropathy [5], it seems that excessive alcohol intake may have been related to the severity of nephropathy in these two patients and might have contributed to development of secondary IgA nephropathy resembling the extensive renal involvement seen in HSP, probably due to the influence of portosystemic shunting.

## Abbreviations

CG: Cryoglobulinemic glomerulopathy
HCV: Hepatitis C virus
IgAN: IgA nephropathy
MGN: Membranous glomerulonephritis
MPGN: Membranoproliferative glomerulonephritis
